# Supplementary material for: The Role of Continuous Glucose Monitoring, Diabetes Smartphone Applications, and Self-Care Behavior in Glycemic Control: Results of a Multi-National Online Survey
Source: J Clin Med. 2019 Jan 17;8(1):109. doi: 10.3390/jcm8010109 (PMC6352012; doi:10.3390/jcm8010109)
Supplement: Supplementary file 1 [file jcm-08-00109-s001.pdf]

Supplementary Figure and Tables

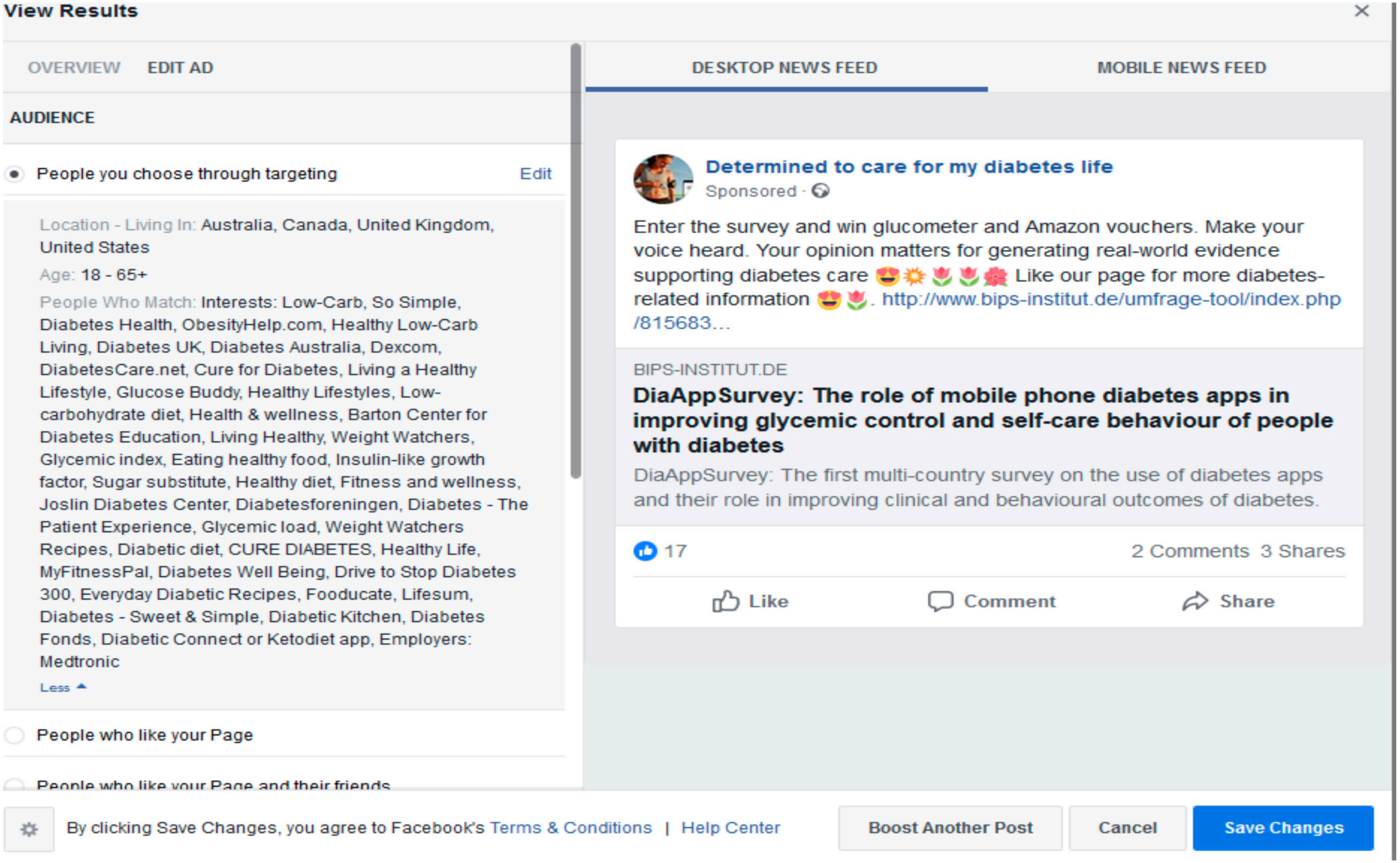

Figure S1: Screenshot of one of the targeted Facebook advertisements and the list of terms used for the advertisement.

Country level differences in factors associated with hyperglycaemia and hypoglycaemia among type 1 and type 2 respondents from the US, Germany and UK.

Table S1. Multinomial logistic regression model of glycemic control in type 1 DM using data from US only respondents.

Variable	Good versus Hyperglycemia in Type 1 Diabetes from US Respondents				Good Versus Hypoglycemia in Type 1 Diabetes from US Respondents			
	RRR	*p*-Value	95% Conf. Interval		RRR	*p*-Value	95% Conf. Interval	
							Lower Limit	Upper Limit
Educational								
Primary to secondary school	1.22	0.563	0.62	2.38	1.07	0.912	0.30	3.78
Polytechnique diploma	2.06	0.102	0.87	4.92	1.22	0.824	0.21	6.91
Sex								
Male	1.47	0.295	0.71	3.03	0.58	0.462	0.14	2.46
Age group								
41 to 60	0.60	0.111	0.32	1.12	1.43	0.554	0.44	4.61
>60	0.89	0.838	0.29	2.75	0.92	0.940	0.10	8.82
Diabetes app use								
Yes	0.90	0.763	0.44	1.83	1.29	0.675	0.39	4.29
**Physical activity**	**0.82**	**0.011**	**0.71**	**0.96**	**1.34**	**0.039**	**1.01**	**1.77**
Specific diet	1.09	0.339	0.91	1.31	0.88	0.422	0.64	1.21
General diet	0.85	0.057	0.72	1.00	0.89	0.476	0.65	1.22
**Blood glucose monitoring**	**0.80**	**0.045**	**0.64**	**0.99**	0.90	0.635	0.57	1.41
On medication								
Yes	0.58	0.661	0.05	6.55	55771.49	0.983	0.00	.
Smoking								
Yes	1.98	0.082	0.92	4.25	3.12	0.090	0.84	11.58
Diabetes Self-management Concern								
High concern	**2.05**	**0.026**	**1.09**	**3.87**	0.73	0.562	0.25	2.14
Foot care	1.02	0.764	0.89	1.17	0.90	0.388	0.70	1.15
CGM user								
Yes	1.14	0.734	0.54	2.40	0.28	0.106	0.06	1.31
_cons	4.14	0.337	0.23	75.18	0.00	0.981	0.00	.

Variables in bold are significant.

Table S2. Multinomial logistic regression model of glycemic control in type 2 DM using data from US respondents.

Variable	Good Versus Hyperglycemia in Type 2 Diabetes from US Respondents			
	**RRR**	***p*-Value**	**95% Conf. Interval**	
Education				
Primary to secondary school	1.37	0.390	0.67	2.81
Polytechnique diploma	1.59	0.302	0.66	3.81
Sex				
Male	1.43	0.398	0.63	3.26
Age group				
**41 to 60**	**3.19**	**0.049**	**1.00**	**10.16**
>60	1.73	0.385	0.50	6.00
Diabetes app use				
Yes	0.61	0.164	0.30	1.23
Physical activity	0.87	0.104	0.74	1.03
Specific diet	1.17	0.105	0.97	1.43
**General diet**	**0.79**	**0.025**	**0.64**	**0.97**
BG monitoring	0.89	0.137	0.77	1.04
Foot care	0.99	0.862	0.85	1.14
On Medication				
Yes	0.85	0.748	0.32	2.28
Smoking				
Yes	1.85	0.184	0.75	4.58
Diabetes Self-management Concern				
**High Concern**	**2.58**	**0.004**	**1.34**	**4.94**
CGM user				
Yes	0.50	0.555	0.05	5.10
_cons	0.65	0.661	0.10	4.41

Variables in bold are significant.

Germany

Table S3. Multinomial logistic regression model of glycemic control in type 1 DM using data from respondents only from Germany.

**Variable**	**Good Versus Hyperglycemia in Type 1 Diabetes Respondents from Germany**				**Good versus Hypoglycemia in Type 1 Diabetes Respondents from Germany**			
	**RRR**	***p*-Value**	**95% Conf. Interval**		**RRR**	***p*-value**	**95% Conf. Interval**	
							**Lower Limit**	**Upper Limit**
Educ								
Primary to secondary school	1.48	0.384	0.61	3.61	0.12	0.055	0.01	1.04
Polytechnique diploma	0.58	0.414	0.16	2.15	0.24	0.284	0.02	3.26
Sex								
Male	0.95	0.902	0.42	2.16	1.11	0.935	0.09	13.28
Age group								
41 to 60	0.96	0.925	0.43	2.15	0.81	0.863	0.08	8.32
>60	0.24	0.241	0.02	2.58	0.00	0.998	0.00	.
Diabetes app use								
Yes	0.57	0.167	0.26	1.26	0.30	0.268	0.03	2.55
Physical activity	1.07	0.455	0.89	1.30	1.64	0.086	0.93	2.90
Specific diet	1.04	0.739	0.84	1.29	0.68	0.235	0.36	1.29
General diet	0.92	0.425	0.74	1.13	0.88	0.673	0.50	1.56
**Blood glucose monitoring**	**0.71**	**0.020**	**0.54**	**0.95**	**0.39**	**0.000**	**0.23**	**0.65**
Foot care	0.93	0.536	0.74	1.17	1.29	0.382	0.73	2.28
On medication								
Yes	2.01	0.250	0.61	6.58	0.81	0.873	0.06	10.97
Smoking								
**Yes**	**2.33**	**0.020**	**1.14**	**4.74**	0.76	0.787	0.10	5.80
Diabetes self-management concern								
**High concern**	**4.55**	**0.000**	**2.08**	**9.95**	2.98	0.312	0.36	24.65
CGM user								
Yes	0.60	0.251	0.25	1.44	0.00	0.989	0.00	.
_cons	0.91	0.940	0.07	11.96	92.18	0.086	0.53	16072.83

Variables in bold are significant.

Table S4. Multinomial logistic regression model of glycemic control in type 2 DM using data from respondents only from Germany.

Variable	Good Versus Hyperglycemia in Type 2 Diabetes Respondents from Germany				Good Versus Hypoglycemia in Type 2 Diabetes Respondents from Germany			
	RRR	*p*-value	95% Conf. Interval		RRR	*p*-value	95% Conf. Interval	
							Lower Limit	Upper Limit
Education								
Primary to secondary school	0.87	0.864	0.18	4.26	0.11	0.189	0.00	2.95
Polytechnique diploma	0.30	0.226	0.04	2.12	0.82	0.908	0.03	24.41
Sex								
Male	0.67	0.383	0.27	1.66	0.51	0.550	0.06	4.58
Age group								
41 to 60	1.34	0.636	0.40	4.52	937,282.50	0.988	0.00	.
**>60**	**5.20**	**0.025**	**1.23**	**21.96**	6,485,043.00	0.986	0.00	.
Diabetes app use								
Yes	0.60	0.395	0.19	1.93	0.51	0.661	0.03	10.20
**Physical Activity**	**0.72**	**0.026**	**0.55**	**0.96**	0.71	0.318	0.37	1.38
Specific diet	1.03	0.862	0.76	1.39	1.10	0.761	0.59	2.07
**General diet**	**0.69**	**0.029**	**0.50**	**0.96**	0.68	0.290	0.34	1.38
Blood glucose monitoring	1.09	0.334	0.92	1.29	1.04	0.881	0.66	1.63
Foot care	0.87	0.192	0.71	1.07	1.28	0.250	0.84	1.97
On medication								
**Yes**	1.12	0.889	0.24	5.21	**0.07**	**0.042**	**0.00**	**0.91**
Smoking								
Yes	0.80	0.678	0.29	2.26	0.77	0.844	0.06	10.64
Diabetes self management concern								
**High concern**	**6.42**	**0.000**	**2.35**	**17.55**	1.22	0.881	0.09	16.93
CGM user								
Yes	2.08	0.578	0.16	27.32	0.00	0.991	0.00	.
_cons	3.52	0.361	0.24	52.04	0.00	0.990	0.00	.

Variables in bold are significant.

United Kingdom

Table S5. Multinomial logistic regression model of glycemic control in type 1 DM using data from UK respondents.

Variable	Good versus Hyperglycemia in Type 1 Diabetes Respondents from UK				Good Versus Hypoglycemia in Type 1 Diabetes Respondents from UK			
	RRR	P-value	95% Conf. Interval		RRR	*p*-Value	95% Conf. Interval	
							Lower Limit	Upper Limit
Education								
**Primary to secondary school**	**6.83**	**0.000**	**2.37**	**19.71**	6.03	0.065	0.89	40.73
Polytechnique diploma	1.94	0.255	0.62	6.04	2.22	0.466	0.26	19.06
Sex								
**Male**	**0.23**	**0.005**	**0.08**	**0.64**	1.80	0.484	0.35	9.43
Age group								
41 to 60	1.08	0.874	0.42	2.76	0.15	0.066	0.02	1.13
>60	2.13	0.435	0.32	14.27	2.05	0.626	0.11	36.69
Diabetes app use								
Yes	1.33	0.569	0.50	3.52	8.60	0.059	0.92	80.13
Physical activity	0.89	0.282	0.71	1.10	0.88	0.540	0.59	1.31
**Specific diet**	**0.70**	**0.015**	**0.52**	**0.93**	1.81	0.065	0.96	3.41
General diet	1.06	0.611	0.84	1.35	0.79	0.280	0.51	1.21
Blood glucose monitoring	1.26	0.142	0.93	1.72	5.16	0.281	0.26	101.54
Foot care	0.97	0.839	0.75	1.27	1.11	0.604	0.74	1.66
On medication								
Yes	0.09	0.134	0.00	2.08	.27	0.504	0.01	12.21
Smoking								
Yes	2.86	0.065	0.94	8.70	.98	0.992	0.08	11.96
Diabetes self-management concern								
High concern	2.41	0.083	0.89	6.51	1.85	0.471	0.35	9.93
CGM user								
**Yes**	**0.08**	**0.004**	**0.01**	**0.46**	0.22	0.147	0.03	1.70
_cons	1.18	0.929	0.03	42.59	0.00	0.157	0.00	394.23

Variables in bold are significant.
